# Study on the high-temperature and aging properties of agricultural waste-modified asphalt based on rheology

**DOI:** 10.1371/journal.pone.0287732

**Published:** 2023-06-29

**Authors:** Renwei Zhang, Zhiyuan Ji

**Affiliations:** 1 College of Civil Engineering, Sanming University, Sanming, China; 2 Key Laboratory of Engineering Material & Structure Reinforement in Fujian Province Colleges (Sanming University), Sanming, China; 3 College of Civil Science and Engineering, Yangzhou University, Yangzhou, China; Shandong University of Technology, CHINA

## Abstract

The modifier of road materials from agricultural waste (AW) as raw material has been widely noticed. Considering the environmental impact of AW treatment and the National policy on the promotion of resource reuse, the feasibility of four AW (namely, bamboo powder, rape straw, corn cob, and wheat straw) for styrene butadiene styrene (SBS) asphalt modification is studied from the properties and mechanism perspectives. Through properties evaluation tests (such as the dynamic shear rheometer, multiple stress creep recovery, and rotating thin film oven test), the influence of four AW and different mixing amounts on the properties of SBS modified asphalt pavement is analyzed from the aspects of high-temperature deformation resistance and anti-aging properties. The results reveal that the four AW can improve the SBS asphalt’s high-temperature deformation resistance and anti-aging properties, among which rape straw has the most significant improvement effect. In addition, through the fourier transform infrared spectroscopy test, the microscopic mechanism of the AW/SBS composite modified asphalt binder is revealed from the functional groups. The analysis shows that the AW is physically mixed with the SBS asphalt binder, which inhibits the growth of sulfoxide groups and the cracking of the SBS modifier during aging.

## Introduction

Asphalt pavement has become a primary, widely used form in high-grade pavement construction owing to its good road performance and ease of construction [1]. However, cracks and other damages seriously affect its service status [2, 3]. The mass proportion of asphalt in asphalt concrete is not large, nonetheless, it has a crucial impact on performance. The commonly used method for enhancing asphalt performance is modifying it using modifiers [4–7]. Among many modifiers, the styrene butadiene styrene (SBS) is capable of forming a physically cross-linked network structure with asphalt under appropriate conditions. Thus, SBS improves the deformation resistance under high temperatures and cracking resistance at low temperatures, which contributes to its wide application [8]. However, with the continuous increase in traffic loads and the frequent occurrence of extreme high-temperature climates, research is focused on improving the performance of SBS asphalt.

In contrast, with global attention to environmental protection policies and resource recycling, road researchers have attempted using recycled waste, which is used as an admixture to study the impact on road materials [9–13]. A large amount of waste is generated in the agricultural production process. The large amounts of agricultural waste (AW) causes severe environmental problems. The resulting AW is often used as a fuel in power plants. For instance, crop residues, such as wheat straw, rape straw, and corn cob, are burned in large quantities to produce ash. In addition, bamboo is a renewable biomass resource and owing to its wide distribution, abundant reserves, and excellent mechanical properties, it has a wide range of applications in China. However, physical and chemical treatments of bamboo also generate waste. Because no proper way has been reported to deal with these crop ashes, some incorrect practices are often adopted, which severely impact the environment and health [14].

According to the data in the FAO report, in China, the annual output of wheat, rape, and corn is 136.95 million tons, 14.71 million tons, and 272.76 million tons, respectively. The production of agricultural products is often accompanied by a large amount of AW, for example, approximately 1300–1400 kg of wheat straw is produced when producing 1 ton of wheat [15]. As previously described, these crops generate significant amounts of AW, which results in environmental problems. Therefore, to overcome this issue, researchers are committed to effectively and rationally disposing of AW. Some scholars have added biomass ash as a modifier to asphalt.

Xue and Arabani et al. [16, 17] studied the modification effect of biomass materials (rice husk ash) as modifiers of asphalt, their results revealed a distinct improvement in the viscosity, and rheological properties of the modified asphalt. Fini et al. [18] studied three types of natural wastes prepared as modifiers (miscanthus pellets, corn stover, and wood pellets), considering the pavement performance and chemical characteristics of modified asphalt binders. Arabani et al. [19] investigated the effect of rice husk ash on asphalt properties by examining the ductility, viscosity, softening point and rheological properties. Moreover, Edeh et al. [20, 21] studied the application of bagasse and peanut husk ash in asphalt pavements. Related literature introduces the modified application of wood biological ash in asphalt. Fareed et al. [22] proposed that three kinds of agricultural waste ash in pavement application scheme to modify asphalt and asphalt concrete, and got good modification effect. Similarly, Ameli et al. [23] explored the feasibility of rice husk ash as an additive agent for asphalt concrete and obtained equally positive conclusions.

In addition to research on ash after the combustion of AW, studies have been conducted on AW treatment using a series of technologies, biomass oil extraction, and asphalt modification using biomass oil as a modifier. Several studies have modified asphalt using different bio-oils and evaluated the modification effect in the laboratory [24, 25]. Raouf et al. [26] modified asphalt with bio-oils extracted from oak wood and reported that the asphalt binder exhibited improved stiffness and high-temperature performance. Han et al. [27] conducted an interesting study, wherein they summarized the research on asphalt modification by plant ash and bio-oil. In addition, they used rice husk ash and bio-oil to compound asphalt, with the hope of obtaining more comprehensive performance. Moreover, they modified the asphalt binder with better high-temperature performance using bio-oil and found that bio-oil positively impacted the crack resistance of the asphalt binder at low temperatures. Castor oil made from castor crop as asphalt modifier was used for research, and it has different modification effects on different kinds of asphalt [28]. The current application of waste bio-oils in roads was summarized by Al-Sabaeei et al. [29]. On the other hand, Zahoor and Zhang et al. introduced in detail the material source and production process of waste bio-oil and the application of waste bio-oil in pavement materials, and emphasized the improvement effect of the asphalt performance [30, 31]. Lv et al. compared the modification effects of five crop bio-oils on rock asphalt and explained the modification mechanism [32].

In summary, previous studies have focused primarily on the modification of asphalt using ash after the combustion of AW and extracting bio-oil through technical treatment. Based on the above investigation, biological ash generally improves the high-temperature performance of asphalt, and the influence of biomass oil prepared by fast pyrolysis, chemical extraction or other methods on asphalt is mainly reflected in the improvement of low-temperature ductility.

However, with global attention to reuse of resources, and the strict requirements for environmental protection, the burning of AW using similar treatment methods is not advocated, particularly in China. Moreover, the treatment of AW combustion is strictly prohibited. In addition, bio-oil’s complex purification and treatment technology occupy more resources and energy, and their application potential is limited. In view of the current problems in the application of AW in asphalt modification and the demand for continuous improvement of the SBS asphalt performance caused by extreme high-temperature climatic conditions. In the context of the sustainable use of resources, the application of more convenient agricultural wastes in SBS asphalt needs to be studied.

In this study, AW was directly processed into powder by physical and mechanical processing. Additionally, its effect as a modifier on the high-temperature properties and anti-aging properties of SBS asphalt were explored. The effects of four types of AW on the rheological properties of SBS asphalt under dynamic shear rheometer (DSR) and multiple stress creep recovery test (MSCR) were investigated. Further the potential of AW as an anti-aging agent was evaluated by comparing the rheological test results of SBS asphalt modified by AW before and after aging. Finally, the modification and anti-aging mechanisms of the four AW on SBS asphalt were revealed from the perspective of functional groups through fourier transform infrared spectroscopy (FTIR) analysis.

The innovations of this study are as follows.

- Explored the feasibility of bamboo, rape straw, corn cob, and wheat straw after physical grinding as modifier of SBS asphalt binder.
- Evaluated the improvement of high temperature and aging resistance of four AW.
- Analyzed the microscopic interaction mechanism between AW and SBS asphalt from the perspective of functional groups.

## Materials and methods

**Fig 1:**
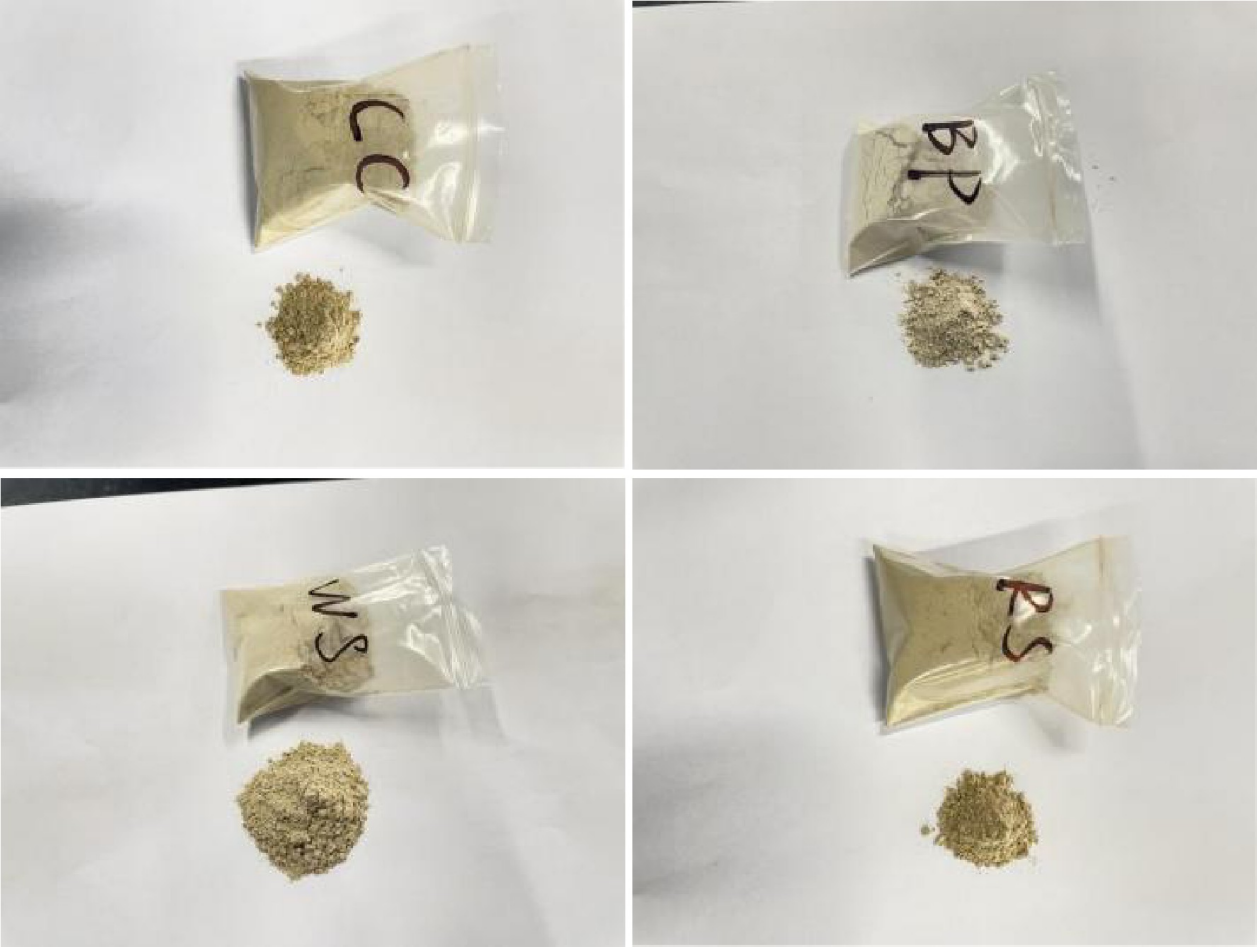
AW powders: (a) CC powder; (b) BP powder; (c) WS powder; and (d) RS powder.

Herein, SBS asphalt is prepared from 70# base asphalt with SBS modifier. The four types of AW that were considered herein, namely yellow bamboo waste (BP), rape straw (RS), corn cob (CC), and wheat straw (WS) (all in yellow powder form), are shown in Fig 1. The base asphalt and SBS modifier were provided by the China Petroleum & Chemical Corporation, and the AW powder was purchased from Fenglian Company. The physical parameters of base asphalt, SBS and four types of Agricultural Waste are shown in Table 1.

**Table 1:** Physical properties of base asphalt, SBS, and agricultural waste.

Material	Physical property	UNIT	Value	Standard
70# base asphalt	Penetration @ 25°C	dmm	70.1	ASTM D5 [33]
	Softening point	°C	49.6	ASTM D36 [34]
	Viscosity @ 135°C	Pa•s	0.5	ASTM D4402 [35]
	Ductility (cm) @ 10°C and 5 cm/min >100	mm	220	ASTM D113-17 [36]
SBS modifier	Appearance	/	Linear leaf	/
	Molecular structure	/	linear	/
	Mass ratio	/	20/80	/
	Tensile strength	Mpa	24.0	/
	Pull the elongation rate	/	730%	
Agricultural Waste (Bamboo powder, Rape straw, Corn cob and Wheat straw)	Grain size	um	45–50	/
	Appearance	/	Yellow powder solid	/
	Purity	/	99.9%	/
	Density	g / cm^3^	2.5–3.5	/

### Preparation process of modified asphalt

The preparation method of composite modified asphalt was introduced in related studies [37–40]. The process of preparing AW/SBS composite modified asphalt binder used was as follows.

1. The base asphalt was heated to 175 ± 5°C, following which 5% (by weight of base asphalt) SBS modifier was added, under this temperature condition, the asphalt has sufficient mobility to better cross-link with the SBS modifier. Next, a high-speed shearing instrument was used for shearing at 175 ± 5°C for 30 min at a speed of 6000 r/min to prepare SBS asphalt for later use.
2. The SBS asphalt was preheated to 175 ± 5°C, the selection of material contents refers to the same type of research [41–44], the different AW powder (5%, 15%, and 15% by weight of SBS asphalt) were added in batches, and a glass rod was used to stir and disperse the powder. Subsequently, the mixture of AW and SBS asphalt was placed in a high-shear machine for high-speed shearing. In order to fully mix AW and SBS asphalt to achieve a better modification effect, the rotation speed and time were set to 6000 r/min and 30 min, respectively.
3. The test asphalt binder samples were numbered to facilitate the analysis and introduction. In this study, the four types of AW modified with SBS asphalt are called AW/SBS asphalt, which does not contradict the name of each asphalt. Table 2 presents the numbering of the different AW/SBS composite modified asphalt binders.

**Table 2:** Asphalt type.

Asphalt type	SBS	AW	Aged asphalt
SBSMA	5%	0	R-SBS
SBP5	5%	5% BP	R-SBP5
SBP10	5%	10% BP	R-SBP10
SBP15	5%	15% BP	R-SBP15
SWS5	5%	5% WS	R-SWS5
SWS10	5%	10% WS	R-SWS10
SWS15	5%	15% WS	R-SWS15
SCC5	5%	5% CC	R-SCC5
SCC10	5%	10% CC	R-SCC10
SCC15	5%	15% CC	R-SCC15
SRS5	5%	5% SR	R-SRS5
SRS10	5%	10% SR	R-SRS10
SRS15	5%	15% SR	R-SRS15

The experiment and test sample information in this study is shown in Table 3.

**Table 3:** Experiment and test sample information.

Experiments	Function	Specification of test sample
DSR	Evaluation of rheological properties	2
MSCR		2
RTFOT	Aging behavior	35 ± 5 g
FTIR	To test the micro-chemical bonds	After softening, 3 g was tested

### Experimental procedures

**Fig 2:**
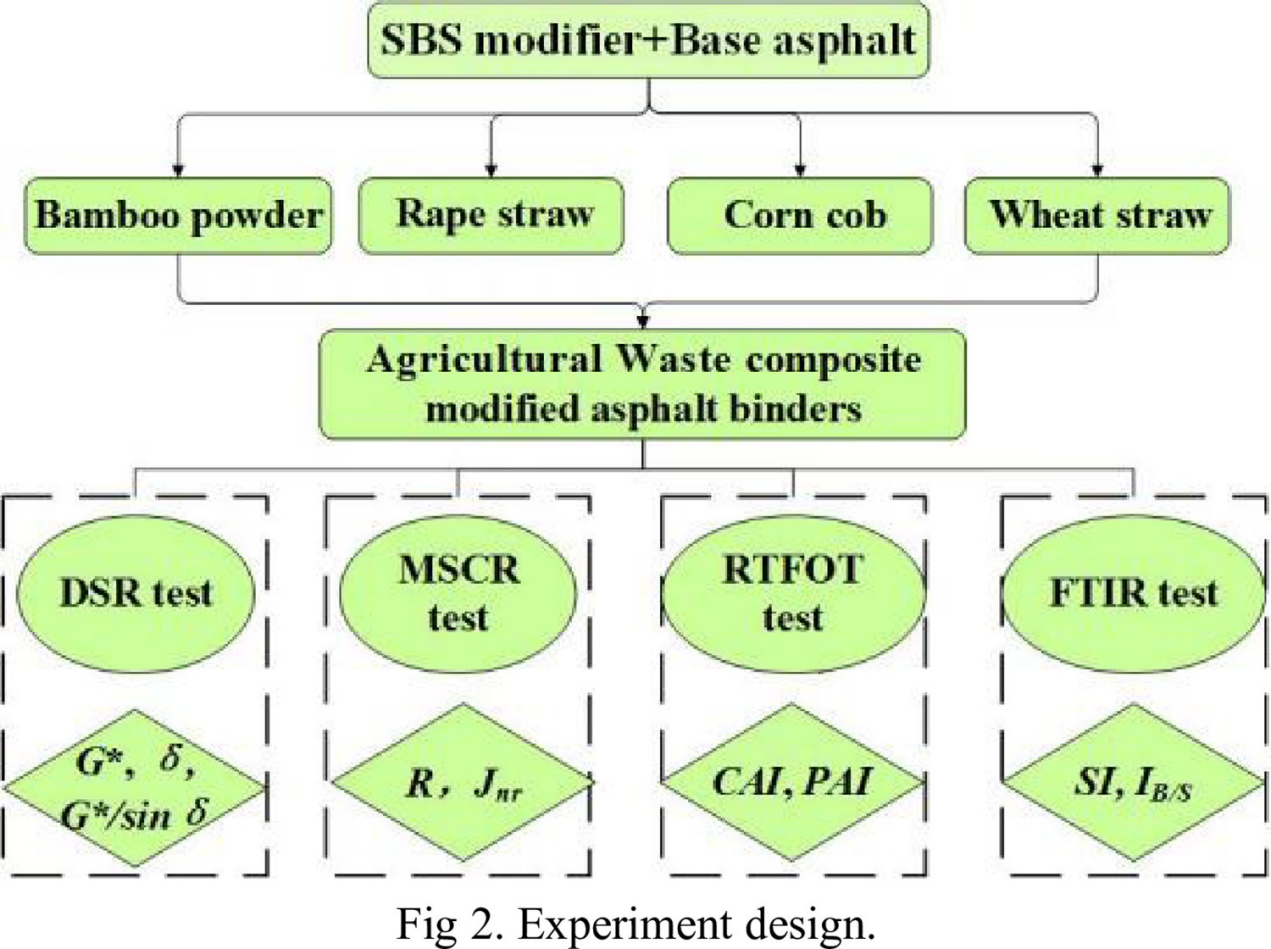
Experiment design.

Fig 2 shows the experimental design of the AW/SBS composite modified asphalt binder in this study.

## Experimental program

### DSR test

The DSR test was used to measure the complex modulus (G*) and phase angle (*δ*) of asphalt, which characterize the shear deformation resistance and viscoelastic properties of asphalt. The test results assisted in characterizing the viscoelastic properties of asphalt under the loading condition of cyclic oscillation. In this study, the examination of asphalt followed the test specification AASHTO T315 [45], and the superpave rutting factor (G*/sinδ) was obtained as an index for evaluating the rutting resistance of the asphalt binder. According to the asphalt properties and laboratory conditions, the test temperature range was set to 52–82°C.

In the report of Bahia et al. [46], it was stated that in loading process, the dissipated energy per cycle is inverse to the G*/sinδ represented in Eq (1).


$$U=\pi \sigma^{2}\left[\frac{1}{G^{*}/\mathrm{Sin}\sigma }\right]$$ (1)

Where *U* is the energy loss per loading cycle (kPa), G* is the complex modulus (kPa), *δ* is the phase angle, and σ is the stress level (kPa).

### MSCR test

The intermittent loading condition is a unique mode of the MSCR test. MSCR test can obtain two important parameters to evaluate the rheological properties of asphalt, which are irrecoverable creep compliance (*J*_*nr*_) and elastic recovery rate (*R*). Their calculations are shown in Eq (2) and Eq (3). The test was performed at two levels of 0.1 kPa and 3.2 kPa, and the test temperature was 64°C. Test operations were performed according to AASHTO T350 [47].


$$J_{nr}\left({\mathrm{kPa}}^{-1}\right)=\frac{\sum_{i=1}^{10}{\left(\frac{\varepsilon_{10}}{\sigma }\right)}_{i}}{10}$$ (2)

$$R(\%)=\frac{\sum_{i=1}^{10}\frac{\varepsilon_{1}-\varepsilon_{10}}{\varepsilon_{1}}}{10}\times 100$$ (3)

Where *R* (%) is the recovery rate, *ε*_1_ is the strain at the end of 1s creep loading, *ε*_10_ is the strain at the end of the recovery period, *J*_*nr*_ is the unrecoverable value (kPa^-1^), and σ is the stress condition (kPa) at each loading stage.

### Rotating thin film oven test

In this study, the asphalt is aged with reference to the method of ASTM D2872 [48], and the aged asphalt can be obtained. The change of rheological properties of the aged asphalt can reflect the anti-aging properties. The anti-aging properties of asphalt binders were quantified using complex modulus aging index (*CAI*) and phase angle aging index (*PAI*) to evaluate the potential of the four AW as anti-aging agents for SBS asphalt [49,50]. The calculation formula for the aging indices is presented in Table 4.

**Table 4:** Aging indices.

Aging indices	Method of calculation
*CAI*	$CAI=\frac{G_{\mathrm{aged}}^{*}}{G_{\mathrm{Unaged}}^{*}}$
*PAI*	$PAI=\frac{\delta_{\mathrm{aged}}}{\delta_{\mathrm{Unaged}}}$

### FTIR test

The change in the macroscopic characteristics of asphalt is reflected at the microscopic level, with the change in the microscopic functional groups as one of the manifestation[51–53]. In general, the functional groups of the material correspond to different characteristic peaks in FTIR tests. Therefore, FTIR was used to investigate the chemical structures of the road materials. The spectrogram of asphalt is helpful to qualitatively analyze the modification mechanism of asphalt, while the functional group index can quantitatively analyze the aging mechanism. The test uses the more convenient ATR mode and the wave number used in the test was set to 500–4000 cm^-1^, and the spectra, including the calculation of peak areas, were processed using EZ OMNIC software.

## Results and discussion

### DSR test analysis

**Fig 3:**
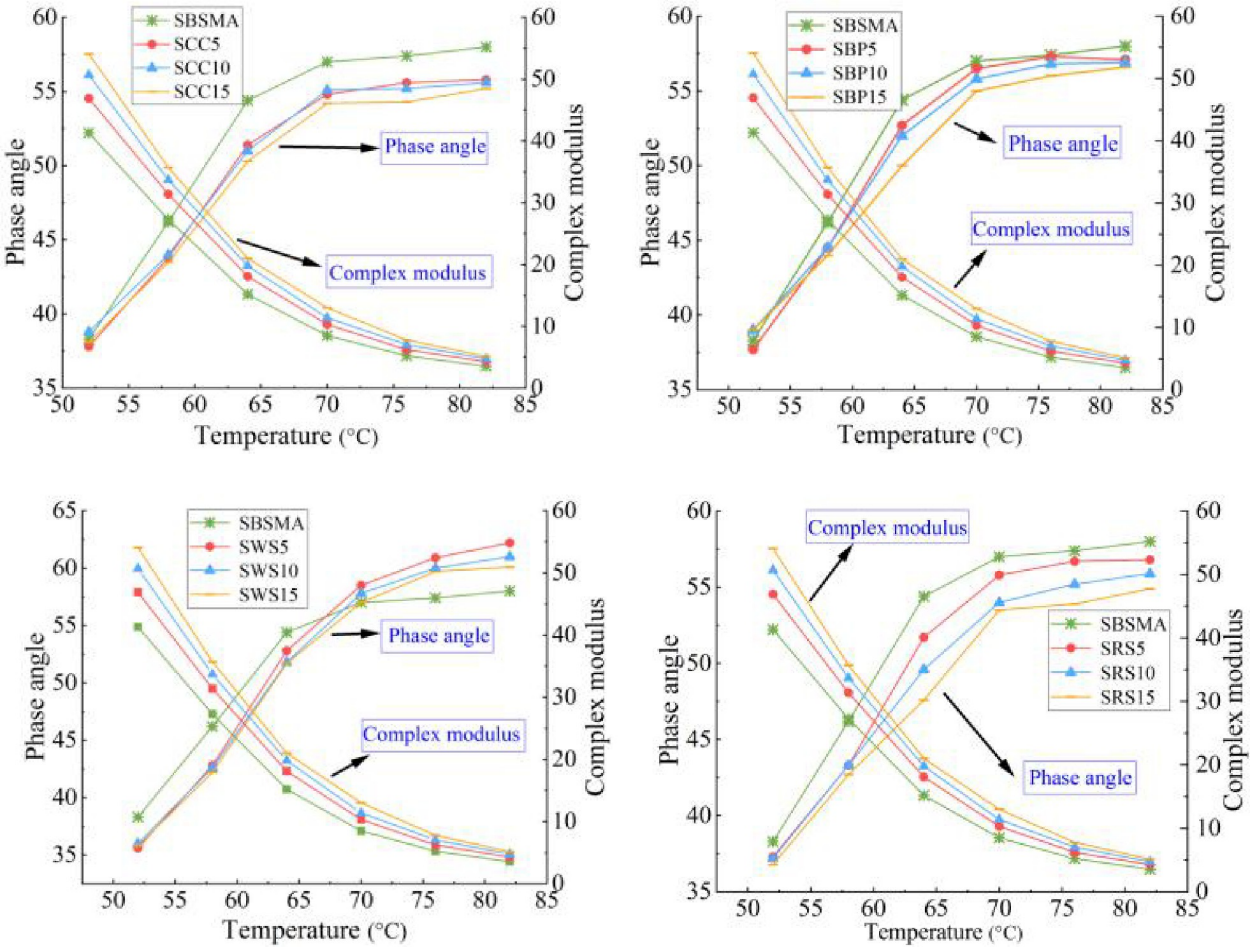
G* and *δ* results of the AW/SBS composite modified asphalt with varying contents: (**a**) Corn cob and SBS asphalt binder (SCC); (b) Bamboo powder and SBS asphalt binder (SBP); (c) Wheat straw and SBS asphalt binder (SWS); and (d) Rape straw and SBS asphalt binder (SRS).

The results of G* and *δ* of the AW/SBS composite modified asphalt with varying contents are shown in Fig 3(A)-3(D). Fig 3 shows that the addition of four different AW led to a distinct increase in the G* of SBSMA at the same test temperature. This effect is evident with increasing incorporation. The G* values of the four different AW/SBS composite modified asphalts all reached the maximum when the AW content was 15%.

The G* value of SBSMA increased, thus indicating improved shear deformation resistance under the action of AW. Moreover, *δ* reflects the viscoelastic response of asphalt materials [54, 55]. Under the same test conditions, the larger the content of BP, RS, and CC, the smaller the *δ* of the SBSMA, this observation indicates that these three types of AW can significantly enhance the elastic response of the SBSMA. In particular, the elastic response of the SRS is most pronounced. Evidently, WS affected the *δ* of SBSMA in different ways at different temperatures. Above 64°C, adding WS to the asphalt increased its *δ*. However, at 52–64°C, WS increased the elastic response of the asphalt, and beyond 64°C, the SBS asphalt was more inclined to a viscous response.

**Fig 4:**
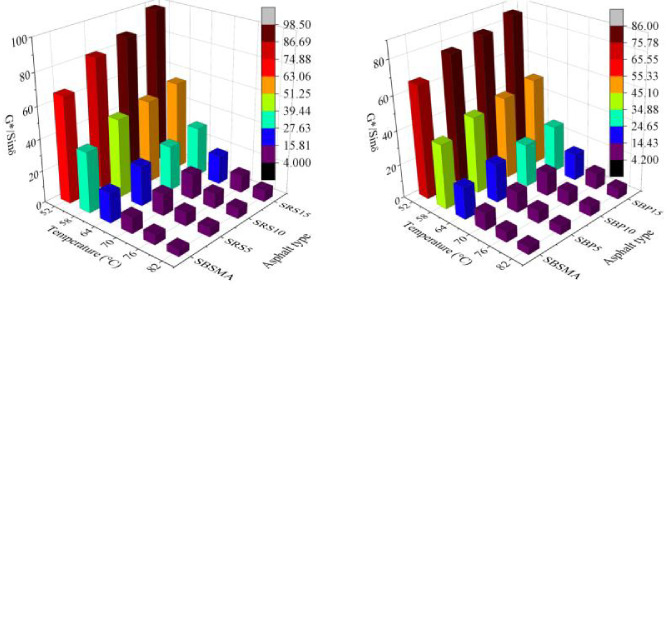
G*/sinδ value of the four AW/SBS composite modified asphalts: (**a**) Corn cob and SBS asphalt binder (SCC); (b) Bamboo powder and SBS asphalt binder (SBP); (c) Wheat straw and SBS asphalt binder (SWS); and (d) Rape straw and SBS asphalt binder (SRS).

Fig 4(a)-4(d) reveal that at the same test temperature, the G*/sinδ values of the four AW/SBS composite modified asphalts exhibited an increasing trend with increasing AW content. In addition, AW most effectively improved the high-temperature rutting resistance of SBSMA at a content of 15%, thus indicating the ability of the four types of AW to enhance SBSMA in terms of rutting resistance under high temperatures. Note that among the four AW modifiers, RS exhibited the most significant improvement in rutting resistance to the high temperature of SBSMA. This may be because of the interaction between AW and SBS asphalt, which renders the SBS asphalt with superior high-temperature rutting resistance.

### MSCR test analysis

In this test, *R*_*0*.*1*_ and *J*_*nr0*.*1*_ represent the test result of asphalt under 0.1 kPa stress conditions. Similarly, *R*_*3*.*2*_ and *J*_*nr3*.*2*_ represent the test result of asphalt under 3.2 kPa stress conditions.

**Fig 5:**
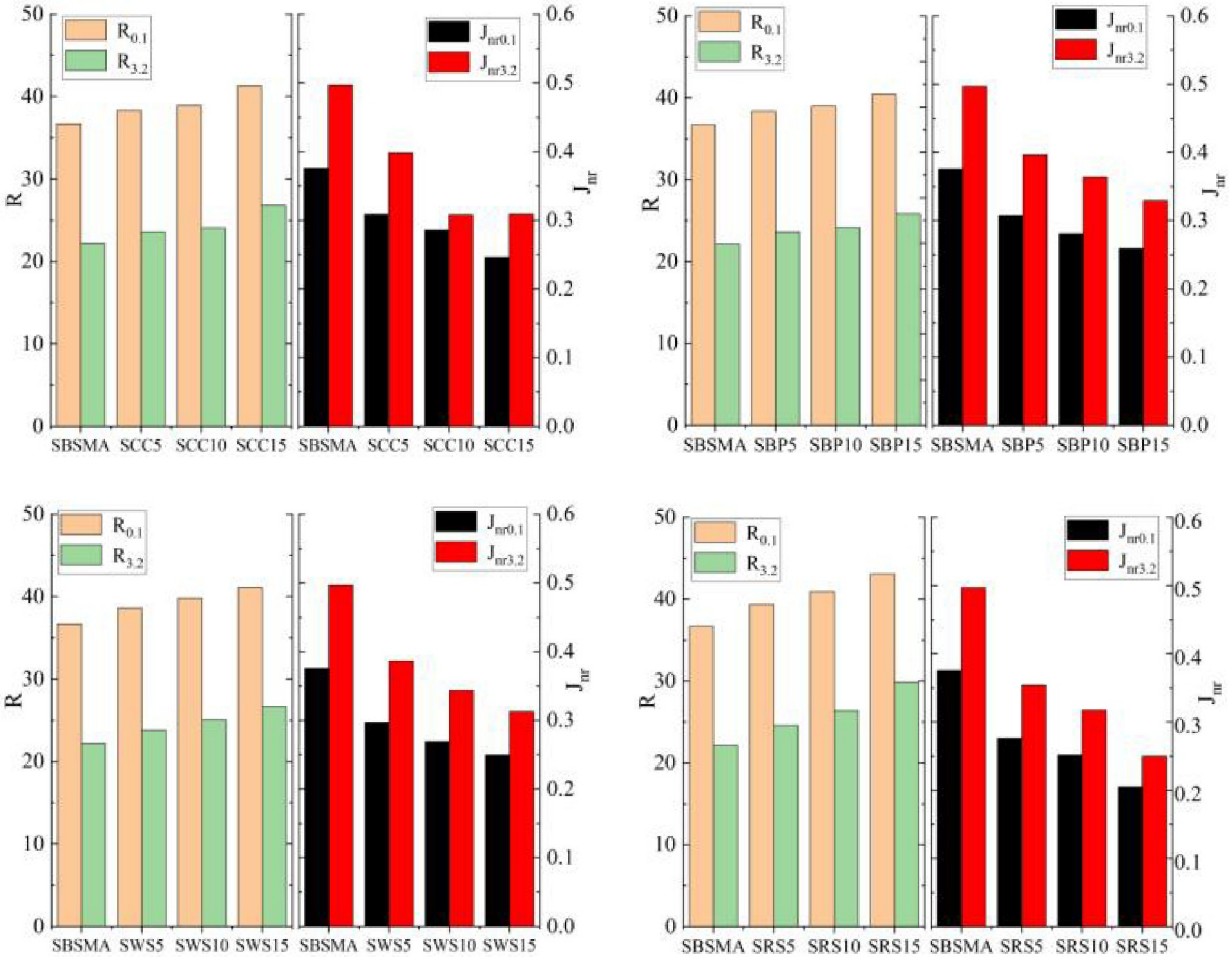
*R* value and *J*_*nr*_ value: (**a**) Corn cob and SBS asphalt binder (SCC); (b) Bamboo powder and SBS asphalt binder (SBP); (c) Wheat straw and SBS asphalt binder (SWS); and (d) Rape straw and SBS asphalt binder (SRS).

Fig 5(a)-5(d) show the effects of the content of AW on SBSMA in terms of the *R* and *J*_*nr*_. Evidently, the addition of AW led to a distinct improvement in *R*, and both the *R*_*0*.*1*_ and *R*_*3*.*2*_ of SBSMA increased as the AW content increases. When the AW content reached 15%, *R*_*0*.1_ and *R*_*3*.*2*_ of AW/SBS composite modified asphalt exhibited the maximum increase. This increase indicates that the proportion of elastic response deformation to total deformation increased after SBSMA modification under stress, which is consistent with the previous analysis of phase angle reduction. It is noteworthy that the *R* of SBSMA increases more significantly under the stress condition of 3.2 kPa. Different stress levels represent traffic load conditions with different magnitudes. The elastic recovery ability under these conditions was more significant, and the modification effect of RS was the most significant. Both *J*_*nr0*.*1*_ and *J*_*nr3*.*2*_ of the SBSMA decreased with increasing content. When the content of AW powder reached 15%, the phases of *J*_*nr0*.*1*_ and *J*_*nr3*.*2*_ of AW/SBS composite modified asphalt exhibited the most distinct decrease compared with SBSMA. Evidently, the smaller the *J*_*nr*_ value, the smaller the irrecoverable deformation caused by the viscous response of the SBSMA, and the better high-temperature deformation resistance [56]. Both MSCR and DSR were tests for evaluating high-temperature properties of asphalt. It was interesting to note that AW affects G*/sinδ, and *R* of SBS asphalt in a similar trend, presumably because the physical properties of the four types of AW are very close to each other.

### Analysis of aging indices

**Fig 6:**
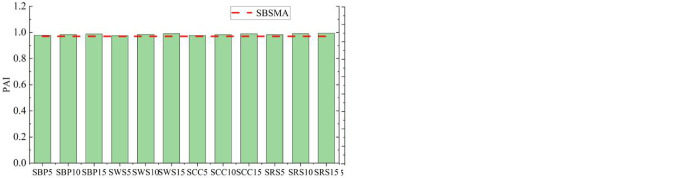
Ageing indices: (a) *CAI* and (b) *PAI* values.

The original data for calculating the aging indices were all calculated at 64°C to facilitate the correlation analysis between the microscopic and the macroscopic rheological indices below. Fig 6 show the *CAI* and the *PAI* of the four AW/SBS modified asphalts, respectively. From Fig 6, the following inferences can be drawn.

1. The complex modulus aging index *CAI* exhibited a decreasing trend with increasing AW content. The *CAI* value of AW/SBS modified asphalt with a content of 15% was the smallest, and the smaller the *CAI* value, the higher the AW/SBS content.
2. The phase angle aging index *PAI* constantly increased as the content increased. The *PAI* value of the AW/SBS modified asphalt with a content of 15% was the largest. A larger *PAI* value indicates improved anti-aging properties.
3. The analysis of the *CAI* and *PAI* aging indices revealed that the four types of AW remarkably improved the anti-aging properties of SBSMA. Among them, SRS modified asphalt exhibited the most robust anti-aging properties.

### FTIR test analysis

The four types of AW remarkably enhanced the high-temperature deformation resistance and anti-aging properties of SBSMA. Consequently, we became interested in exploring the microscopic mechanism of the action of AW on SBSMA. Thus, the modification and anti-aging mechanisms of the four types of AW and SBSMA were explored.

**Fig 7:**
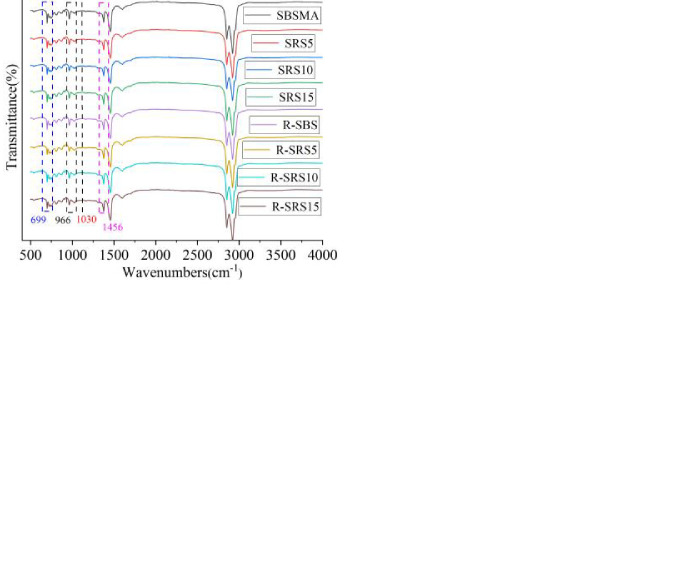
FTIR spectrum of SBSMA and AW/SBS modified asphalt: (**a**) Corn cob and SBS asphalt binder (SCC); (b) Bamboo powder and SBS asphalt binder (SBP); (c) Wheat straw and SBS asphalt binder (SWS); and (d) Rape straw and SBS asphalt binder (SRS).

Fig 7(A)–7(D) show the infrared spectra of SBSMA and AW/SBS composite modified asphalt. Evidently, the infrared spectrum of the SBSMA exhibited characteristic peaks at 2921, 2851, 1599, 1456, 1376, 1030, 862, 810, and 722 cm^− 1^. The absorption peaks at 2921 cm^-1^ and 2851 cm^-1^ corresponded to the characteristic peaks of aliphatic *C-H* stretching vibration, that at 1599 cm^-1^ corresponded to the stretching vibration of aromatic *C-C*, those at 1456 cm^-1^ and 1376 cm^-1^ were the bending vibration absorption peak of *C-H*, and the stretching vibration of sulfoxide group (*S = O*) at 1030 cm^-1^, respectively. The absorption peak at 700 cm^-1^–862 cm^-1^ was the characteristic peak of the *C-H* bending vibration on the benzene ring. In addition, characteristic peaks of the polymer were observed at 699 cm^-1^ and 966 cm^-1^, of which 699 cm^-1^ corresponded to the vibration absorption peak of the mono-substituted benzene ring of polystyrene (*PS*) in SBS, whereas that at 966 cm^-1^ was the polybutylene twisting vibrational absorption peak of *C = C* bonds in polybutadiene (*PB*).

If the chemical reaction occurs in the modification process of asphalt, the chemical structure of the molecule will change under the strong interaction, and the infrared spectrum of asphalt after the modification will also change compared with the original asphalt spectrum. Consequently, the appearance of new characteristic absorption peaks is the most common phenomenon. A comparison of the infrared spectra of SBSMA and SBP/SBS, SWC/SBS, SCC/SBS, and SRS/SBS composite modified asphalt binders revealed that the peak phases of the characteristic absorption peaks were close to each other. The AW powder is dispersed and mixed inside the SBSMA to increase the viscosity of the SBSMA. The close physical properties of the four AW powder, such as particle size and density, indicates that the addition of AW did not alter the molecular properties of SBSMA. Hence, the spectra of the modified asphalt binders are very similar to the original SBSMA binder [57–59]. In addition, no new characteristic absorption peak appeared. Thus, the modification process between the four types of AW and SBSMA can be regarded as a physical modification.

To further explore the influence of AW on the microchemical structure of SBSMA aging behavior, the characteristic peak area parameters of the sulfoxide group (*S = O*) were selected for calculation and analysis. The sulfoxide index (*SI*) represents the change in sulfoxide functional groups and has been widely used in asphalt aging research [60]. In this study, *SI* was used as the quantitative analysis index to explore anti-aging properties of SBS asphalt. It was calculated as using the Eq (4), as follows.

$$SI=\frac{A_{S=O}}{A}$$ (4)

where *A*_*S = O*_ is the absorption peak area centered at 1030 cm^-1^, and *A* is the absorption peak area centered at 1456 cm^-1^.

Under the action of aging conditions, the polar groups in the asphalt that are sensitive to high temperature and oxygen conditions changed. The sulfide in the asphalt participated in the oxidation reaction, thereby causing the content of sulfoxide groups, located at 1030 cm^-1^, to change. Consequently, the characteristic peak area was affected. R-SBS and aged AW/SBS exhibited larger characteristic peak areas of sulfoxide groups at 1030 cm^-1^ compared with unaged asphalt.

*I*_*B/S*_ is a reference index used to characterize the polymer content in the SBS asphalt. It is the ratio of the peak area centered at 966 cm^-1^ to that centered at 699 cm^-1^. SBSMA under aging conditions, the 966 cm^-1^ peak changes significantly, while the 699 cm^-1^ peak is more stable, so this ratio can reflect the polymer content. Detailed studies on asphalt aging have been conducted [61]. The calculation of *I*_*B/S*_ is shown in Eq (5), as follows.

$$I_{B/S}=PB/PS=\frac{A_{966\mathrm{cm}}{}^{–1}}{A_{699\mathrm{cm}}{}^{–1}}$$ (5)

where A_966_ cm^-1^ is the absorption peak area centered at 966 cm^-1^, and A_699_ cm^-1^ refers to the area centered at 699 cm^-1^.

Compared with SBSMA, R-SBS exhibited an enhanced absorption peak of the sulfoxide group (*S = O*) at 1030 cm^-1^, and the peak area increased.

**Fig 8:**
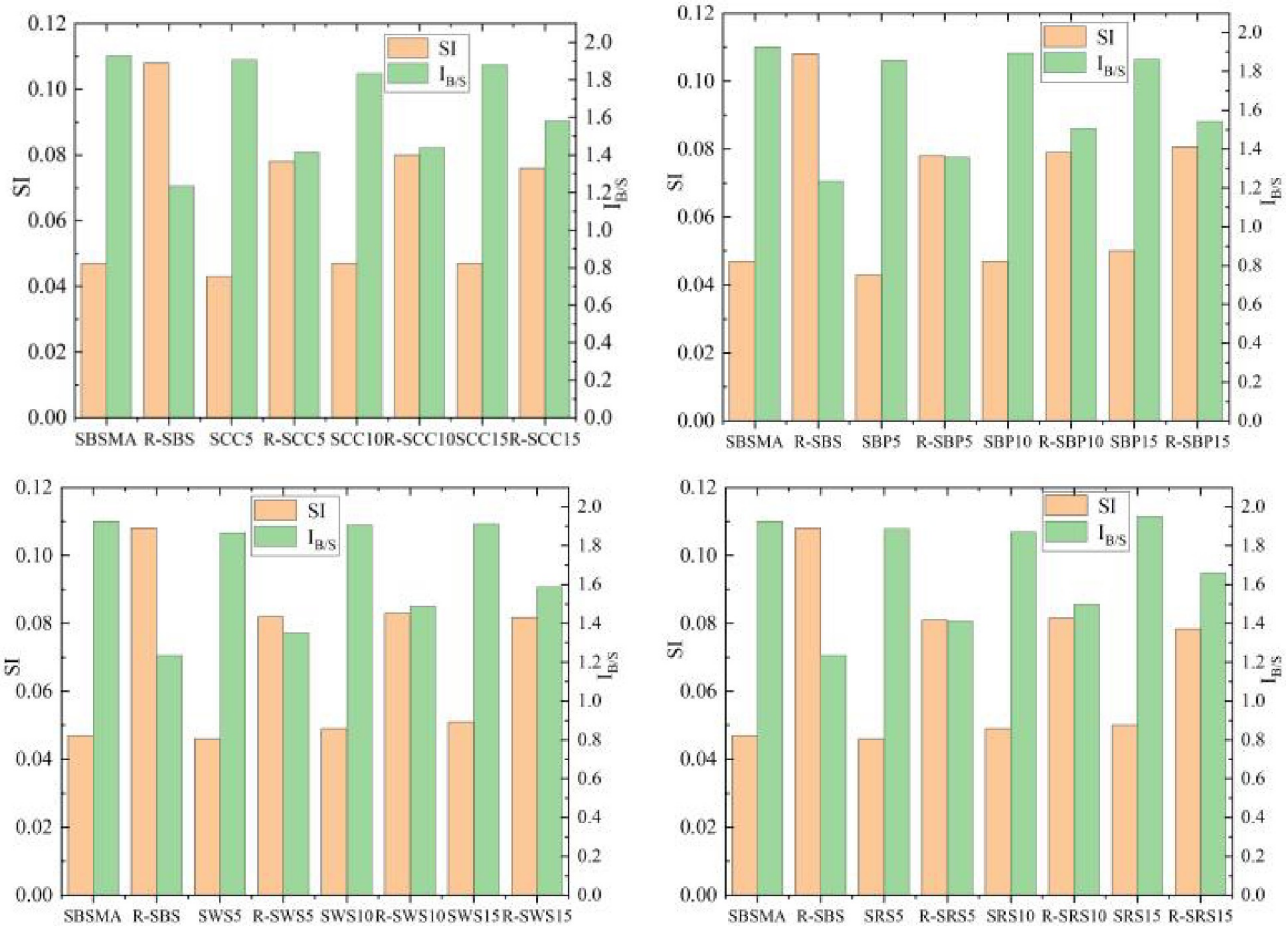
*SI* and *I*_*B/S*_ results of SBSMA and the four AW/SBS composite modified asphalts and their aged samples: (**a**) Corn cob and SBS asphalt binder (SCC); (b) Bamboo powder and SBS asphalt binder (SBP); (c) Wheat straw and SBS asphalt binder (SWS); and (d) Rape straw and SBS asphalt binder (SRS).

The *SI* and *I*_*B/S*_ results of SBSMA, and the four AW/SBS composite modified asphalts and their aged samples are shown in Fig 8.

First, the *SI* analysis of the asphalt system revealed no distinct difference between the *SI* values of the four AW/SBS composite modified asphalts and the *SI* values of SBSMA. This is due to the changes in the sulfoxide group are caused by the oxidation reactions, while the change in polymer content is only related to the polymer additive content of SBS asphalt itself. The addition of the AW did not affect their chemical bonds. After RTFOT, the *SI* of the SBSMA and four AW/SBS modified asphalts increased. In contrast, the AW/SBS modified asphalt exhibited a smaller increase in *SI* relative to SBSMA, and the larger the AW content, the smaller the increase in *SI*. The increases in *SI* of SBP15, SWS15, SCC15, SRS15, and SBSMA were 61.2%, 60.2%, 61.7%, 56.6%, and 129.79%, respectively. The *SI* increases for the four types of AW/SBS composite modified asphalt with the content in the AW content reaching 15% were much smaller than those of SBSMA, which indicates that the four types of AW weakened the sensitivity of the *S = O* to the thermal-oxygen environment and inhibited the growth of the *S = O*.

Second, from the perspective of polymer analysis of *I*_*B/S*_, the SBS modifier had the same content in the four AW/SBS composite modified asphalt and SBSMA. After aging, *I*_*B/S*_ values decreased for all five asphalt binders. The decreases in *I*_*B/S*_ of SBP15, SWS15, SCC15, SRS15, and SBSMA were 17.19%, 16.95%, 15.9%, 14.96%, and 35.88%, respectively. The *I*_*B/S*_ values of AW/SBS composite modified asphalt with an AW content of 15% exhibited a smaller decrease compared with the SBSMA, thus indicating that the addition of AW can effectively inhibit the cracking behavior of polymer under aging conditions. When SBSMA was aged, AW relieved the growth of asphalt sulfoxide groups and the cracking of polymer under the aging effect of SBSMA through the mutual adsorption of sensitive segments in asphalt and polymer, thereby improving the anti-aging properties.

### Correlation analysis

Previous studies have shown that the properties of asphalt is affected when it is affected by modifiers or aging conditions. This is because the sensitive functional groups in asphalt are highly sensitive to modifiers or aging conditions, these sensitive functional groups change under external factors, thereby affecting the asphalt performance. In the previous FTIR analysis, *SI* was selected as the reference index to explore microscopic mechanism of AW/SBS composite modified asphalt. This section introduces a new index *ΔSI*, whose calculation method is shown in Eq (6).


$$\Delta SI=SI_{\mathrm{aged}}-SI_{\mathrm{Unaged}}$$ (6)

Table 5 lists the sulfoxide group index *SI* and high-temperature rheological indices of SBSMA and AW/SBS composite modified asphalt (G*, *δ*, G*/sinδ, *R*_*0*.1_, *R*_*3*.*2*_, *J*_*0*.*1*_, and *J*_*3*.*2*_).

**Table 5:** High-temperature rheological indices and *SI*.

Asphalt type	*SI*	G*/ sinδ	*δ*	G*	*R*_*0*.*1*_	*R*_*3*.*2*_	*J*_*0*.*1*_	*J*_*3*.*2*_
SBSMA	0.047	15.2	54.4	66.60	36.68	22.17	0.3757	0.4968
SBP15	0.050	21.0	50.0	85.96	40.42	25.81	0.2595	0.3294
SCC15	0.047	22.0	51.7	88.55	41.12	26.63	0.2494	0.3128
SRS15	0.050	23.0	50.3	89.14	41.28	26.82	0.2460	0.309
SWS15	0.051	23.0	47.6	99.00	43.96	29.86	0.2050	0.2500

**Fig 9:**
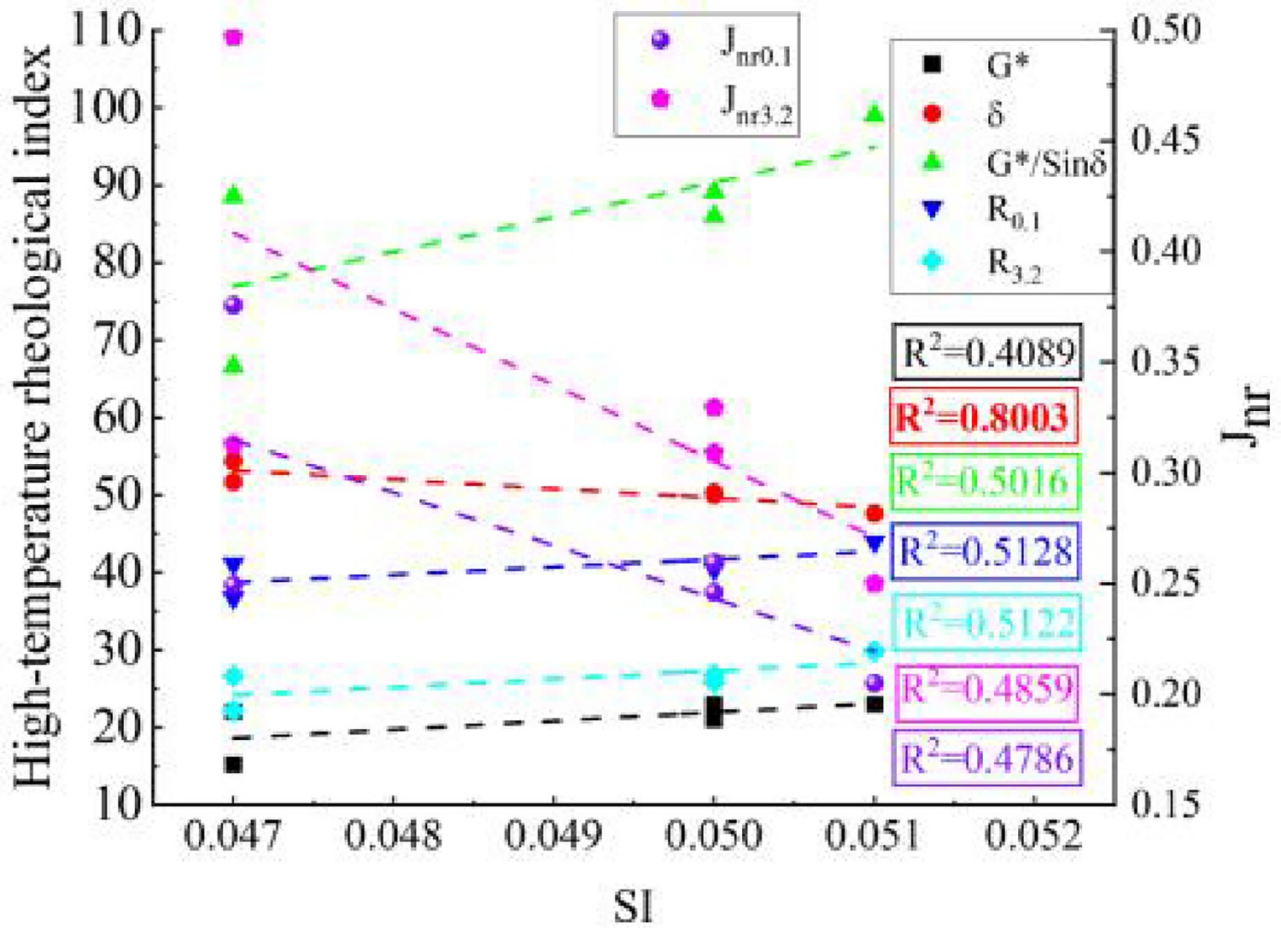
Correlation analysis between *SI* and high-temperature rheological indices.

Fig 9 shows the association of the high-temperature rheological indices with the *SI*. Among the high-temperature rheological indices, the correlation between *δ* and *SI* was the best (R^2^ = 0.8003), which suggests that the phase angle can not only serve as an index for inversely evaluating the elastic response of asphalt materials but also can be used as a reference to reflect the *SI* content of AW/SBS composite modified asphalt. Table 6 lists the *ΔSI*, *R-I*_*B/S*_ and aging indices (*CAI* and *PAI*) of the SBSMA and AW/SBS composite modified asphalt (in the analysis of this section, the 15% content of AW/SBS modified asphalt with the most significant modification effect was selected).

**Table 6:** ΔSI, R-I_B/S_ and ageing indices (CAI and PAI).

Asphalt type	*ΔSI*	*R-I*_*B/S*_	*CAI*	*PAI*
SBSMA	0.0610	1.235	1.56	0.97
SBP15	0.0306	1.542	1.13	0.988
SCC15	0.0290	1.582	1.12	0.989
SRS15	0.0283	1.66	1.08	0.994
SWS15	0.0307	1.587	1.11	0.99

Relevant studies have highlighted that SBSMA has a complex aging process, and two processes of polymer cracking and base asphalt hardening occur simultaneously. In the FTIR analysis part of this study, *SI* and *R-I*_*B/S*_ were used to analyze the mechanism of aging reaction. The relationship between polymer cracking reactions during aging of AW/SBS modified asphalt and high-temperature rheological properties of SBSMA before and after aging, as well as the relationship between the hardening reactions of asphalt systems and high-temperature rheological properties of SBSMA before and after aging, respectively, deserve further investigation. The aging indices (*CAI* and *PAI*) obtained based on high-temperature rheological indices were compared with *ΔSI* to explore the association of the aging indices with the sulfoxide group in the asphalt system, the aging indices (*CAI* and *PAI*) and *R-I*_*B/S*_ obtained from the high-temperature rheological indices were used to analyze the association of the aging indices with polymer cracking.

**Fig 10:**
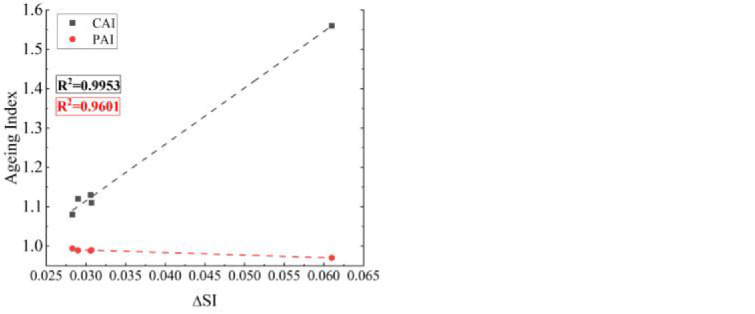
Correlation analysis.

The relationship between aging indices and *R-I*_*B/S*_ is presented Fig 10(A). The correlation between the *PAI* (R^2^ = 0.9988) and *R-I*_*B/S*_ was the best. During the aging process, SBS modifier degradation is an important factor that affects the SBSMA performance. The larger the *R-I*_*B/S*_, the higher the SBS modifier content after aging, and the lower the cracking degree.

The relationship between aging indices and *ΔSI* is illustrated in Fig 10(B). The best correlation between the *CAI* (R^2^ = 0.9953) and *ΔSI* represents the change in the G* before and after aging. Smaller *CAI* values indicate that the asphalt has better aging resistance, and therefore the shear deformation resistance is less affected by the aging test. *ΔSI* shows the change in the sulfoxide group content before and after asphalt aging, which corresponds to the analysis of the sulfoxide group content above, thus indicating that *CAI* can be used to reflect the change in sulfoxide group content in the aging reaction of the AW/SBS composite-modified asphalt.

## Conclusion

In this study, four types of AW, namely BP, WS, CC, and RS, were used to modify SBSMA. The effect of different contents of AW on the rheological properties of SBSMA was evaluated by DSR and MSCR tests. The modified asphalt binder was subjected to thermo-oxidative aging to explore its aging characteristics. Finally, the microscopic action mechanism of AW on SBSMA was analyzed using a microscopic FTIR test from the perspective of functional groups. The study following four main conclusions were drawn.

1. The analysis of DSR test results revealed that the four types of AW contributed to an obvious improvement in the high-temperature properties of SBSMA, and the effect was significant as the content of AW increased.
2. The *R* value and *J*_*nr*_ value under different stress in the MSCR test revealed that the four types of AW could enhance the rutting resistance of SBSMA, and improvement effect was more evident under the condition of heavy traffic. Among them, the modification of SBSMA by RS was the most effective.
3. The test results of RTFOT revealed that adding AW improved the anti-aging properties of SBSMA, thus indicating that the four types of AW have the potential to be used as anti-aging agents for SBSMA. Similarly, the modification of SBSMA by RS was the most significant.
4. The qualitative analysis of the FTIR spectrum revealed that the modification process of the four types of AW and SBSMA was performed in the form of physical blending. Furthermore, quantitative analysis of the functional group index revealed that AW could simultaneously inhibit the growth of *SI* in SBSMA under thermo-oxidative aging conditions and inhibit the cracking of the SBS modifier, thereby enhancing the anti-aging ability of SBSMA.

In this study, AW/SBS modified asphalts have better high-temperature properties and anti-aging properties compared with SBSMA, which provides a reference for the construction of agricultural waste asphalt pavement in perennial high-temperature areas.

## Supporting information

S1 File Data. This file includes all the experiment data of the asphalt binders.
(DOCX)
