# Supplementary material for: Study on the high-temperature and aging properties of agricultural waste-modified asphalt based on rheology
Source: PLoS One. 2023 Jun 29;18(6):e0287732. doi: 10.1371/journal.pone.0287732 (PMC10309637; doi:10.1371/journal.pone.0287732)
Supplement: S1 File — This file includes all the experiment data of the asphalt binders. (DOCX) [file pone.0287732.s001.docx]

**Date**

1. **Materials and Methods**

Table 1. Physical properties of base asphalt, SBS, and Agricultural Waste

| Material | Physical property | UNIT | Value | Standard |
| --- | --- | --- | --- | --- |
| 70# base asphalt | Penetration @ 25 °C | dmm | 70.1 | ASTM D5 [33] |
|  | Softening point | °C | 49.6 | ASTM D36 [34] |
|  | Viscosity @ 135°C | Pa•s | 0.5 | ASTM D4402 [35] |
|  | Ductility (cm) @ 10°C and 5 cm/min >100 | mm | 220 | ASTM D113-17 [36] |
| SBS modifier | Appearance | / | Linear leaf | / |
|  | Molecular structure | / | linear | / |
|  | Mass ratio | / | 20/80 | / |
|  | Tensile strength | Mpa | 24.0 | / |
|  | Pull the elongation rate | / | 730% |  |
| Agricultural Waste (Bamboo powder、  Rape straw  、Corn cob  and Wheat straw) | Grain size | um | 45-50 | / |
|  | Appearance | / | Yellow powder solid | / |
|  | Purity | / | 99.9% | / |
|  | Density | g / cm^3^ | 2.5-3.5 | / |

1. **DSR test analysis**

Table 2. Phase angle (*δ*) and complex modulus (*G**) of SCC modified asphalts

| Temperature | *δ* | | | | *G** | | | |
| --- | --- | --- | --- | --- | --- | --- | --- | --- |
|  | SBS | SCC5 | SCC10 | SCC15 | SBS | SCC5 | SCC10 | SCC15 |
| 52 °C | 38.3 | 37.8 | 38.8 | 38.1 | 41.3 | 46.7 | 50.2 | 55 |
| 58 °C | 46.2 | 43.8 | 44 | 43.5 | 27.3 | 31.4 | 33.5 | 36.6 |
| 64 °C | 54.4 | 51.4 | 51 | 50.3 | 15.2 | 18.4 | 20 | 23 |
| 70 °C | 57 | 54.8 | 55.1 | 54.2 | 8.5 | 10.7 | 12 | 13.8 |
| 76 °C | 57.4 | 55.6 | 55.2 | 54.3 | 5.23 | 6.73 | 7.5 | 8.5 |
| 82 °C | 58 | 55.8 | 55.6 | 55.2 | 3.54 | 4.57 | 4.99 | 5.32 |

Table 3. Phase angle (*δ*) and complex modulus (*G**) of SBP modified asphalts

| Temperature | *δ* | | | | *G** | | | |
| --- | --- | --- | --- | --- | --- | --- | --- | --- |
|  | SBS | SBP5 | SBP10 | SBP15 | SBS | SBP5 | SBP10 | SBP15 |
| 52 °C | 38.3 | 37.7 | 39 | 39 | 41.3 | 46.9 | 50.7 | 54.1 |
| 58 °C | 46.2 | 44.5 | 44.5 | 44 | 27.3 | 31.4 | 33.7 | 35.7 |
| 64 °C | 54.4 | 52.7 | 52 | 50 | 15.2 | 18.1 | 19.8 | 21 |
| 70 °C | 57 | 56.5 | 55.8 | 55 | 8.5 | 10.3 | 11.4 | 13 |
| 76 °C | 57.4 | 57.3 | 56.8 | 56 | 5.23 | 6.23 | 7.06 | 7.8 |
| 82 °C | 58 | 57.1 | 56.9 | 56.6 | 3.54 | 4.28 | 4.75 | 5.15 |

Table 4. Phase angle (*δ*) and complex modulus (*G**) of SWS modified asphalts

| Temperature | *δ* | | | | *G** | | | |
| --- | --- | --- | --- | --- | --- | --- | --- | --- |
|  | SBS | SWS5 | SWS10 | SWS15 | SBS | SWS5 | SWS10 | SWS15 |
| 52 °C | 38.3 | 35.6 | 36 | 35.8 | 41.3 | 45.6 | 49.2 | 51.8 |
| 58 °C | 46.2 | 42.8 | 42.6 | 42.2 | 27.3 | 32.2 | 34.9 | 36.8 |
| 64 °C | 54.4 | 52.8 | 51.8 | 51.7 | 15.2 | 19.1 | 20.8 | 22 |
| 70 °C | 57 | 58.5 | 57.8 | 57 | 8.5 | 10.7 | 11.8 | 13.6 |
| 76 °C | 57.4 | 60.9 | 60 | 59.7 | 5.23 | 6.43 | 7.34 | 8.01 |
| 82 °C | 58 | 62.2 | 61 | 60.1 | 3.54 | 4.12 | 4.79 | 5.28 |

Table 5. Phase angle (*δ*) and complex modulus (*G**) of SRS modified asphalts

| Temperature | *δ* | | | | *G** | | | |
| --- | --- | --- | --- | --- | --- | --- | --- | --- |
|  | SBS | SRS5 | SRS10 | SRS15 | SBS | SRS5 | SRS10 | SRS15 |
| 52 °C | 38.3 | 37.3 | 37.2 | 36.8 | 41.3 | 49.8 | 53.1 | 57.2 |
| 58 °C | 46.2 | 43.3 | 43.3 | 42.7 | 27.3 | 33.6 | 35.6 | 37.6 |
| 64 °C | 54.4 | 51.7 | 49.6 | 47.6 | 15.2 | 19.6 | 21.4 | 23 |
| 70 °C | 57 | 55.8 | 54 | 53.5 | 8.5 | 11.2 | 12.7 | 14.7 |
| 76 °C | 57.4 | 56.7 | 55.2 | 53.9 | 5.23 | 6.94 | 7.8 | 8.8 |
| 82 °C | 58 | 56.8 | 55.9 | 54.9 | 3.54 | 4.68 | 5.31 | 5.8 |

Table 6. Superpave rutting factor (*G*/sinδ*) of SCC modified asphalts

| Temperature | SBSMA | SCC5 | SCC10 | SCC15 |
| --- | --- | --- | --- | --- |
| 52 °C | 66.6 | 76.4 | 80.11 | 89.14 |
| 58 °C | 37.9 | 45.3 | 48.22 | 53.17 |
| 64 °C | 18.7 | 23.5 | 25.74 | 29.29 |
| 70 °C | 10.1 | 13 | 14.63 | 17.01 |
| 76 °C | 6.21 | 8.16 | 9.13 | 10.41 |
| 82 °C | 4.27 | 5.53 | 6.04 | 6.47 |

Table 7. Superpave rutting factor (*G*/sinδ*) of SBP modified asphalts

| Temperature | SBSMA | SBP5 | SBP10 | SBP15 |
| --- | --- | --- | --- | --- |
| 52 °C | 66.6 | 76.7 | 80.6 | 85.96 |
| 58 °C | 37.9 | 44.9 | 48.1 | 51.4 |
| 64 °C | 18.7 | 22.7 | 25.1 | 27.4 |
| 70 °C | 10.1 | 12.3 | 13.7 | 15.8 |
| 76 °C | 6.21 | 7.57 | 8.45 | 9.41 |
| 82 °C | 4.27 | 5.1 | 5.67 | 6.17 |

Table 8. Superpave rutting factor (*G*/sinδ*) of SWS modified asphalts

| Temperature | SBSMA | SWS5 | SWS10 | SWS15 |
| --- | --- | --- | --- | --- |
| 52 °C | 66.6 | 78.3 | 83.7 | 88.55 |
| 58 °C | 37.9 | 47.4 | 51.56 | 54.78 |
| 64 °C | 18.7 | 24 | 26.47 | 28.03 |
| 70 °C | 10.1 | 12.6 | 13.94 | 16.22 |
| 76 °C | 6.21 | 7.36 | 8.47 | 9.28 |
| 82 °C | 4.27 | 4.66 | 5.47 | 6.09 |

Table 9. Superpave rutting factor (*G*/sinδ*) of SRS modified asphalts

| Temperature | SBSMA | SRS5 | SRS10 | SRS15 |
| --- | --- | --- | --- | --- |
| 52 °C | 66.6 | 82.1 | 87.83 | 98.49 |
| 58 °C | 37.9 | 49 | 51.91 | 55.74 |
| 64 °C | 18.7 | 25 | 28.1 | 31.15 |
| 70 °C | 10.1 | 13.5 | 15.7 | 18.29 |
| 76 °C | 6.21 | 8.3 | 9.5 | 10.89 |
| 82 °C | 4.27 | 5.6 | 6.41 | 7.09 |

1. **MSCR test analysis**

Table 10. Elastic recovery rate (*R*) and irrecoverable creep compliance (*J_nr_*) of AW/SBS composite modified asphalts.

| Asphalt type | *R_0.1_* | *R_3.2_* | *J_nr0.1_* | *J_nr3.2_* |
| --- | --- | --- | --- | --- |
| SBSMA | 36.68 | 22.17 | 0.3757 | 0.4968 |
| SCC5 | 38.33 | 23.58 | 0.3088 | 0.3984 |
| SCC10 | 38.91 | 24.05 | 0.2858 | 0.308 |
| SCC15 | 41.28 | 26.82 | 0.246 | 0.309 |
| SBP5 | 38.37 | 23.6 | 0.3075 | 0.3967 |
| SBP10 | 39 | 24.12 | 0.281 | 0.3639 |
| SBP15 | 40.42 | 25.81 | 0.2595 | 0.3294 |
| SWS5 | 38.62 | 23.81 | 0.297 | 0.3862 |
| SWS10 | 39.81 | 25.09 | 0.2692 | 0.3438 |
| SWS15 | 41.12 | 26.63 | 0.2494 | 0.3128 |
| SRS5 | 39.38 | 24.57 | 0.276 | 0.354 |
| SRS10 | 40.93 | 26.4 | 0.2515 | 0.3174 |
| SRS15 | 43.06 | 29.86 | 0.205 | 0.25 |

1. **Analysis of aging indices**

Table 11. Complex modulus aging index (*CAI*) and phase angle aging index (*PAI*) of AW/SBS composite modified asphalts.

| Asphalt type | *CAI* | *PAI* |
| --- | --- | --- |
| SBSMA | 1.56 | 0.97 |
| SCC5 | 1.29 | 0.976 |
| SCC10 | 1.23 | 0.982 |
| SCC15 | 1.12 | 0.989 |
| SBP5 | 1.28 | 0.978 |
| SBP10 | 1.22 | 0.983 |
| SBP15 | 1.13 | 0.988 |
| SWS5 | 1.26 | 0.975 |
| SWS10 | 1.16 | 0.985 |
| SWS15 | 1.11 | 0.99 |
| SRS5 | 1.16 | 0.983 |
| SRS10 | 1.1 | 0.99 |
| SRS15 | 1.08 | 0.994 |

1. **FTIR test analysis**

Table 12. *SI* and *I_B/S_* of AW/SBS composite modified asphalt before and after aging.

| Asphalt type | *SI* | *I_B/S_* |
| --- | --- | --- |
| SBSMA | 0.047 | 1.926 |
| R-SBS | 0.108 | 1.235 |
| SCC5 | 0.043 | 1.905 |
| R-SCC5 | 0.078 | 1.413 |
| SCC10 | 0.047 | 1.834 |
| R-SCC10 | 0.08 | 1.439 |
| SCC15 | 0.047 | 1.881 |
| R-SCC15 | 0.076 | 1.582 |
| SBP5 | 0.043 | 1.856 |
| R-SBP5 | 0.078 | 1.356 |
| SBP10 | 0.047 | 1.893 |
| R-SBP10 | 0.079 | 1.506 |
| SBP15 | 0.05 | 1.862 |
| R-SBP15 | 0.0806 | 1.542 |
| SWS5 | 0.046 | 1.867 |
| R-SWS5 | 0.082 | 1.352 |
| SWS10 | 0.049 | 1.905 |
| R-SWS10 | 0.083 | 1.489 |
| SWS15 | 0.051 | 1.911 |
| R-SWS15 | 0.0817 | 1.587 |
| SRS5 | 0.046 | 1.886 |
| R-SRS5 | 0.081 | 1.410 |
| SRS10 | 0.049 | 1.870 |
| R-SRS10 | 0.0815 | 1.496 |
| SRS15 | 0.05 | 1.952 |
| R-SRS15 | 0.0783 | 1.660 |

1. **Correlation analysis**

Table 13. High-temperature rheological indices and *SI*

| Asphalt type | *SI* | *G*/ sinδ* | *δ* | *G** | *R_0.1_* | *R_3.2_* | *J_0.1_* | *J_3.2_* |
| --- | --- | --- | --- | --- | --- | --- | --- | --- |
| SBSMA | 0.047 | 15.2 | 54.4 | 66.60 | 36.68 | 22.17 | 0.3757 | 0.4968 |
| SBP15 | 0.050 | 21.0 | 50.0 | 85.96 | 40.42 | 25.81 | 0.2595 | 0.3294 |
| SCC15 | 0.047 | 22.0 | 51.7 | 88.55 | 41.12 | 26.63 | 0.2494 | 0.3128 |
| SRS15 | 0.050 | 23.0 | 50.3 | 89.14 | 41.28 | 26.82 | 0.2460 | 0.309 |
| SWS15 | 0.051 | 23.0 | 47.6 | 99.00 | 43.96 | 29.86 | 0.2050 | 0.2500 |

Table 14. *ΔSI*, *R-I_B/S_* and ageing indices（*CAI* and *PAI*）

| Asphalt type | *ΔSI* | *R-I_B/S_* | *CAI* | *PAI* |
| --- | --- | --- | --- | --- |
| SBSMA | 0.0610 | 1.235 | 1.56 | 0.97 |
| SBP15 | 0.0306 | 1.542 | 1.13 | 0.988 |
| SCC15 | 0.0290 | 1.582 | 1.12 | 0.989 |
| SRS15 | 0.0283 | 1.66 | 1.08 | 0.994 |
| SWS15 | 0.0307 | 1.587 | 1.11 | 0.99 |

Table 15. Correlation results

| Index | *G*/ sinδ* | *δ* | *G** | *R_0.1_* | *R_3.2_* | *J_0.1_* | *J_3.2_* |
| --- | --- | --- | --- | --- | --- | --- | --- |
| *SI* | 0.5016 | 0.8003 | 0.4089 | 0.5128 | 0.5122 | 0.4786 | 0.4859 |

Table 16. Correlation results

| Index | *CAI* | *PAI* |
| --- | --- | --- |
| *ΔSI* | 0.9953 | 0.9601 |
| *R-I_B/S_* | 0.9716 | 0.9988 |
